# Livestock Inspectors

**Published:** 1898-07

**Authors:** 


### CANADIAN LIVESTOCK INSPECTORS.

Canada employs a large number of livestock inspectors located throughout the several provinces. The following-named veterinarians are employed under salary: J. A. Armstrong, Nelson, B. C.; M. C. Baker, Montreal, Quebec; A. Brown, Point Edward (Sarnia), Ontario; J. A. Couture, Quebec, P. Q.; Victor Daubigny, Montreal, Quebec; J. H. Frinck, St. John, N. B.; Wm. Jakeman, Halifax, N. S.; Jos. Kime, Chatham, Ontario; A. A. Leckie, Charlottetown, P. E. I.; C. McEachran, Montreal, Quebec; D. McEachran, Chief Veterinary Inspector, Montreal, Quebec; A. E. Moore, Montreal, Quebec; G. W. Orchard, Windsor, Ontario; M. B. Perdue, Kingsville, Ontario; W. H. Pethick, Central Bedeque, P. E. I.; P. A. Robinson, Emerson, Manitoba; Prof. Andrew Smith, Toronto, Ontario; B. A. Sugden, Montreal, Quebec; J. R. Thorne, Wallaceburg, Ontario; veterinary surgeons of the Northwest mounted police.

Two hundred and thirteen other veterinarians fill the rôle of inspectors, being paid by fees. The certificates of all these inspectors are accepted by the Department of Agriculture at Washington in the livestock exportations to this country: D. H. Ackerill, Belleville; James Airth, Chatsworth; James Armitage, Kincardine; Frederick Armstrong, Fergus; J. G. Alexander, Mono Road; Thomas Arrall, Caledonia, Ontario.

J. W. Bland, Vancouver, B. C.; Jas. A. Bean, Gananoque, Ontario; Josephus Baily, Barrie, Ontario; E. P. Ball, Stanstead Plain, Quebec; John W. Barr, Newton; George Beacom, Harriston; G. H. Belaire, Pembroke; J. E. Blackall, Clinton; William Brady, Tilsonburg; O. E. Brandreth, Cayuga; S. E. Boulter, Niagara Falls; David Bone, Chesley; W. F. Broad, Lindsay; C. J. Brodie, Claremont; Fred Charles Brown, Rodney; James A. Buchan, L'Original; J. C. Burk, Blenheim; W. H. Burns, King P. O.; C. W. Bell, Kingston; H. Bradshaw, Napanee; Frank Bryant, Sunderland, Ontario; Samuel Burkholder, Gore Bay, Manitoulin Island; P. T. Bowlby, Tweed, Ontario; M. G. Blanchard, Victoria, B. C.

A. B. Campbell, Berlin; W. J. Cunnington, Thornbury; Geo. S. Cavin, Princeton; James Chesney, Hensall; J. L. Clarke, Stratford; W. F. Clarke, Goderich; Richard C. Coates, Thamesville; William Colcleugh, Mount Forest; W. A. R. Coleman, Jarvis; R. Colgan, St. Catharine's; Richard Coghlan, Wyoming; R. Colthurst, Bothwell; J. M. Colvin, Teeswater; Hugh Cooper,

Toronto; D. Coristine, Oil Springs; J. J. Coutts, Galt; M. C. Crawforth, Whitby; James Cruickshanks, Heathcote; C. M. Culbertson, Meaford; D. Currie, Coldwater; W. H. Clapp, Dresden, Ontario; S. A. Coxe, Brandon, Manitoba; J. C. Christie, Greenwood, B. C.

Frank Daly, Sutton West, Ontario; W. Dann, Cranton, Quebec; Henry Domville, Woodstock, N. B.; John Donoghue, St. Thomas, Ontario; O. H. Duncombe, Waterford, Ontario; Charles Dwyer, Sutton, Quebec.

C. E. Eaid, Simcoe; Louis Eckert, Sebringville; John James Elliott, Clifford; John Engel, Milverton; W. Ewing, Keeswick, Ontario.

Wesley H. Farrow, Wroxeter; Robert Fawns, Parry Sound; John Fife, Palmerston; A. G. Fortune, Walkerton; C. D. Fortune, Shelburne; J. W. Fasken, Paris; R. H. Fortune, Ayton; F. Fisher, Carleton Place; H. W. Fry, Dunnville, Ontario.

William Gibb, St. Mary's; Orr Graham, Port Perry; James Grant, Bervie; W. J. Gerrow, Woodville; D. Geddes, Lucknow; Robert F. Golden, Windsor; Edward Golden, Elora; A. T. Goldie, Corunna; Robert Grant, Paisley; John Grieve, Seaforth, Ontario; J. O. Guy, St. Jean, Quebec; R. L. Graham, Schomberg, Ontario.

D. Hamilton, Harriston; Phil Harold, Tavistock; E. W. Heighway, London; D. Henderson, Glencoe; F. Hewitt, Hanover; John Howie, Guelph; C. R. Howell, Melbourne; D. Hood, Alliston; W. H. Huck, Mildmay; H. D. Hurd, Toronto, Ontario; H. R. Hunter, Coaticock, Quebec.

J. D. Irvine, Vankleek Hill, Ontario; J. J. Irwin, Waterloo, Quebec; George Ireland, Guelph, Ontario.

A. E. James, Ottawa; John C. Johnston, Chesley; W. J. Johnston, Brigden; Spencer Jupp, Petrolia, Ontario.

W. Kenny, Lindsay; R. T. Kidd, Listowell; B. W. Kitley, Sharon, Ontario.

Adam Landreth, Bright; Jos. Lambertus, Teeswater; Major Lloyd, Newmarket, Ontario; C. Little, Winnipeg, Manitoba; William Lawson, Dundas, Ontario; A. M. Livingston, Melita, Manitoba.

John W. Manchester, Apohaqui, N. B.; James Mayhew, Cookstown; H. S. Manhard, Smith's Falls; C. R. Mitchell, Owen Sound; A. S. Morrison, Chesterville; W. J. Morgan, Kingston; J. H. Morse, Shedden, Ontario; L. Mulligan, Shawville, Quebec; William Mole, Toronto; Malcolm Munro, Lancaster; J. A.

Mobray, Oshawa; Arch McArthur, Elmvale; Donald McAlpine, Brockville; D. McArthur, Ailsa Craig; D. McColl, Parkhill; Thomas McConnell, Toronto, Ontario; A. McCormick, Ormstown, Quebec; D. McCuaig, Moncton, N. B.; James McDonald, Summerside, P. E. I.; John Henry McDonald, Wiarton, Ontario; R. P. McGahey, Kemptville; J. McGillicuddy, Watford, Ontario; John McCurdy, Granby, Quebec; David McIntosh, Brucefield; James M. McKay, Malten; W. H. McLaren, Highgate; J. H. McLean, Paplar Hill; W. B. McMicken, Plattsville, Ontario; J. L. McMillan, Charlottetown, P. E. I.; W. A. McNeely, Brooklin; Arch McTaggart, Arthur; R. H. McKenna, Picton; D. D. McNaughton, Loggan; J. C. McMurtry, Arnprior, Ontario.

F. S. Nixon, Dundalk, Ontario.

E. C. Oliver, Stayner; S. Ottowelt, Feversham; W. H. Orme, Ilderton, Ontario.

J. D. Paxton, Ayr; J. N. Perdue, Blyth; James Pickel, Drayton; W. H. Pickering, Forest; J. W. Porter, Burford; John Prudham, Alvinston; Thomas Purvis, Belleville; John Perdue, Orangeville, Ontario.

J. Hugo Reed, Guelph; W. H. Riddell, Orangeville; W. H. Richardson, Essex; F. L. Robinson, Peterborough; C. L. Robson, Myrtle; J. A. Roe, Attwood; E. S. Rogers, Thessalon; H. A. Rose, Selkirk; W. B. Rowe, Ridgetown; S. C. Rudd, Woodstock, Ontario; A. E. Ramsay, Eden Mills, Ontario.

James Stewart, Nairn; A. W. Shields, West Toronto; Walter Shillinglaw, Mitchell; E. P. Smith, Cambray; Wm. Stevens, St. Mary's; W. W. Stewart, Toronto, Ontario; J. A. Stevenson, Morden, Manitoba; E. Stevenson, Bradford; Parker Stevenson, Norwood; J. G. Stewart, Brantford; Donald A. Stewart, Ivan; Thomas Stirling, New Hamburg; W. W. Stork, Brampton; William Sweet, Exeter; William Breckhill Sein, Elmira; Charles L. Smith, Brantford, Ontario; W. H. Smith, Carman, Manitoba.

W. Tanner, Mount Forest; E. Tennant, Lucan; J. H. Tennant, London; M. H. Teneyky, Hamilton; Fred Thomas, Tara; A. E. Thompson, Warkworth; J. H. Tooley, Delhi, Ontario; A. W. Tracy, Cookshire, Quebec; J. I. Tellier, St. Hyacinthe, Quebec; Thomas Thacker, Renfrew; Joseph Thomson, Orillia, Ontario.

Alexander Wannan, Oshawa; John D. Warwick, Brussels; Adam Watson, Coburg; J. B. White, Port Hope; T. H. Wilkinson, Port Elgin; R. W. Wilkinson, Drumbo; E. T. Williams,

Sunderland; J. M. Wilkinson, Ripley; John Wilson, Wingham; J. P. Whitehead, Napier; J. P. Whitehead, Strathroy; A. C. Wolfe, Durham; Jacob Wagner, Tavistock or Hickson; F. A. Walsh, Yarker, Ontario.

Robert Young, Bowmanville, Ontario.

